# Sliding Window Analyses for Optimal Selection of Mini-Barcodes, and Application to 454-Pyrosequencing for Specimen Identification from Degraded DNA

**DOI:** 10.1371/journal.pone.0038215

**Published:** 2012-05-29

**Authors:** Stephane Boyer, Samuel D. J. Brown, Rupert A. Collins, Robert H. Cruickshank, Marie-Caroline Lefort, Jagoba Malumbres-Olarte, Stephen D. Wratten

**Affiliations:** 1 Department of Ecology, Faculty of Agriculture and Life Sciences, Lincoln University, Lincoln, New Zealand; 2 Bio-Protection Research Centre, Lincoln University, Lincoln, New Zealand; Brigham Young University, United States of America

## Abstract

DNA barcoding remains a challenge when applied to diet analyses, ancient DNA studies, environmental DNA samples and, more generally, in any cases where DNA samples have not been adequately preserved. Because the size of the commonly used barcoding marker (COI) is over 600 base pairs (bp), amplification fails when the DNA molecule is degraded into smaller fragments. However, relevant information for specimen identification may not be evenly distributed along the barcoding region, and a shorter target can be sufficient for identification purposes. This study proposes a new, widely applicable, method to compare the performance of all potential ‘mini-barcodes’ for a given molecular marker and to objectively select the shortest and most informative one. Our method is based on a sliding window analysis implemented in the new R package SPIDER (Species IDentity and Evolution in R). This method is applicable to any taxon and any molecular marker. Here, it was tested on earthworm DNA that had been degraded through digestion by carnivorous landsnails. A 100 bp region of 16 S rDNA was selected as the shortest informative fragment (mini-barcode) required for accurate specimen identification. Corresponding primers were designed and used to amplify degraded earthworm (prey) DNA from 46 landsnail (predator) faeces using 454-pyrosequencing. This led to the detection of 18 earthworm species in the diet of the snail. We encourage molecular ecologists to use this method to objectively select the most informative region of the gene they aim to amplify from degraded DNA. The method and tools provided here, can be particularly useful (1) when dealing with degraded DNA for which only small fragments can be amplified, (2) for cases where no consensus has yet been reached on the appropriate barcode gene, or (3) to allow direct analysis of short reads derived from massively parallel sequencing without the need for bioinformatic consolidation.

## Introduction

DNA barcoding has been increasingly used for both species discovery and specimen identification. This approach is based on amplification and sequencing of DNA regions that are informative at the species level. For Metazoa, the mitochondrial cytochrome oxidase subunit 1 (COI) gene is recognised as the standard DNA barcode and the basis for the Barcode Of Life project [1]. However, other molecular markers such as 12 S, 16 S, 18 S, 28 S, ITS1, ITS2 and COII, are commonly used in the same way to serve similar purposes. Therefore, following Valentini et al. [2], we adopt the term DNA barcoding *sensu lato* to encompass research using any of these alternative markers. Ideally, DNA barcoding studies use fresh or preserved tissue samples as sources of DNA. However, in many situations this is not possible and degraded DNA must be used instead. This is the case for diet analyses [3], ancient DNA studies [4], specimen identification from environmental DNA samples [2], and more generally for any DNA sample that has not been adequately preserved. The main difficulty associated with amplifying poorly preserved or degraded DNA is the disintegration of the DNA molecule into short fragments [4].

Studies of degraded DNA preferentially target mitochondrial genes due to their higher number of copies per cell, and therefore their greater amplification success than single-copy nuclear genes [5]. Despite this, amplifying degraded DNA remains a challenge [6]. The length of the most commonly used barcoding marker (COI) is more than 600 bp. If the DNA molecules are broken down into fragments that are smaller than this then it will not be possible to amplify this region since none of these fragments will contain the binding sites for both primers [4]. However, information relevant for specimen identification may not be evenly distributed along the barcoding region, and a shorter target or ‘mini-barcodes’ can often be sufficient for identification purposes [7]. No general method currently exists to objectively compare the performance of all potential ‘mini-barcodes’ and to select the best one for a given set of taxa. This has led previous studies focusing on the standard DNA barcoding region to rely on non-optimised mini-barcodes with a comprehensive but not absolute resolution at the species level [8]–[11].

In diet analyses, pieces of prey tissue isolated from a predator's gut often contain enough non-degraded prey DNA for PCR amplification [12]–[14]. However, heavily digested prey DNA diffused in the gut ‘soup’, or remaining in the predator's faeces is difficult to isolate and amplify [15], which may lead to some prey species being overlooked. Similarly, in environmental samples, well preserved DNA is preferentially amplified by conventional primers while any degraded DNA is likely to remain undetected. Another important issue inherent to environmental samples and diet analyses is the presence of genetic material from several species in a single mixed sample. Individual sequencing of all species in a mixed DNA sample can, however, be achieved through massively parallel sequencing methods, such as 454-pyrosequencing. Pyrosequencing technology is capable of simultaneously detecting many thousands of different sequences in a mixed sample, without the need for sub-cloning [16].

We propose a new method for selecting short but informative DNA fragments for specimen identification from degraded DNA samples, and for sequencing and identifying all the species present in a mixed DNA sample. Our method is based on a newly developed R package SPIDER (SPecies IDentity and Evolution in R) that provides customisable, user-friendly functions for calculating a diverse range of summary statistics useful for DNA barcoding, taxonomy and analysis of species-level evolution [17]. We tested our method on earthworm DNA that had been degraded through digestion by the carnivorous landsnail *Powelliphanta augusta* (Mollusca: Pulmonata: Rhytididae). The 16 S rDNA region was selected to create an earthworm DNA library because previous research has highlighted the value of this molecular marker over COI for earthworm taxonomy at genus and species levels [18]–[20]. In addition, this marker is usually composed of alternating stretches of variable and conserved sequences, which are ideal for specimen identification and the design of internal primers. Using the sliding window analysis implemented in SPIDER, we selected the shortest fragment of 16 S rDNA that contains sufficient information for accurate and reliable specimen identification. Corresponding primers were then designed and used to amplify degraded earthworm (prey) DNA from landsnail (predator) faeces. Because *P. augusta* may feed on many different species of earthworms, PCR products may contain mixed DNA that is not compatible with conventional Sanger sequencing. Therefore, 454-pyrosequencing was used to sequence DNA from each predated species.

## Materials and Methods

### Ethics statement

Animal handling and sampling methods were conducted according to relevant national and international guidelines. All necessary permits were obtained from the New Zealand Department of Conservation.

### Sample collection

A large proportion of the original habitat of *P. augusta* has recently been lost to opencast coal mining at Stockton mine (on the West Coast of New Zealand's South Island) [21]. Prior to mining, in October–November 2006 and May 2007, snails found in the field were placed in individual clean plastic containers and any faecal strings produced within 24 hours were retained and stored in ethanol (95%) at −20°C. Because previous studies on other rhytidid snails have highlighted the importance of earthworms in their diet [22], an earthworm inventory was conducted in 2008 and 2009. About 1,500 earthworms were collected from the remainder of the original habitat of *P. augusta*, as well as from surrounding disturbed and undisturbed habitats by excavation and hand sorting of 300 soil blocks (20 cm×20 cm×20 cm) [23]. All earthworms found in these areas were New Zealand endemic species (Oligochaeta: Megascolescidae and Acanthodrilidae).

### Earthworm DNA libraries

A total of 139 earthworm specimens representative of all sampled morphotypes were selected to build a DNA library of the species potentially predated by *P. augusta*. DNA extractions were performed on earthworm muscle using the Axygen Biosciences extraction kit (animal tissue spin protocol). Universal invertebrate 16 S rDNA primers (LR-J-12887 and LR-N-13398) [24] were used to amplify a ∼500 bp fragment of DNA (see [25] for full protocol).

Molecular analyses revealed the presence of 15 distinct clades with a minimum divergence of 4%, representing 15 putative species, yet to be described [25]. Because intra-clade variation was generally low, the earthworm DNA library was built using a single representative sequence from each clade. Reference sequences were aligned with MAFFT version 6 [26] and pruned to a 430 bp section (including indels) that runs from positions 11,736 to 12,118 of the *Lumbricus terrestris* (Oligochaeta: Lumbricidae) mitochondrial genome sequence [27].

### Sliding window analysis

The statistical programming language R is a powerful, flexible and free environment for the analysis of a wide range of data, including nucleotide sequences [28]. We used the sliding window function *slideAnalyses* in the R package SPIDER [17] version 1.0–5 (http://spider.r-forge.r-project.org/) to determine the shortest informative window that best discriminated the reference earthworm sequences. This function extracts all possible windows of a chosen size in a DNA alignment, and performs a variety of distance and tree-based measures on each window.

As an effective specimen identification tool relies on each species having a unique DNA profile, within each window, genetic distance values greater than zero can permit effective differentiation. It is also advantageous to retain phylogenetic information so that species not represented in the DNA library can be correctly assigned at a higher taxonomic level. For this reason, windows that best represent the topology given by the full 430 bp alignment are favoured. To this effect, we selected the shortest informative window by considering the following distance matrix and tree-based criteria: (1) the proportion of zero pairwise non-conspecific distances in the matrix; and (2) the proportion of identical clades shared between the neighbour-joining tree derived from the full 430 bp dataset, and those derived from each window. Windows with no zero non-conspecific distances and a proportion of identical clades greater than 85% for shallow nodes (i.e. nodes tipwards of the median node depth) were considered as highly informative because they allow accurate specimen identification, and provide a good representation of the tree topology for the full-dataset. Windows of 25, 50, 100, 150, 200 and 250 bp were analysed and compared to determine the shortest highly informative window. Conserved regions on either side of the selected window were then investigated with the aim of designing degenerate primers that amplify only the DNA of New Zealand endemic earthworms (Oligochaeta: Megascolecidae and Acanthodrilidae). These are referred to as ‘group-specific primers’ below. The specificity of these primers was tested on lumbricid earthworms, which are not native to New Zealand and according to a recent comprehensive survey [29], do not occur in the snail's habitat. The non-target species tested were: *Eisenia fetida*, *E. andrei* and *Lumbricus terrestris*. This specific approach aimed to prevent the amplification of DNA from non-target taxa, which may occur in snail faeces. This includes DNA from the snail itself, DNA from other invertebrate species (especially parasites), and bacterial DNA.

### 454-pyrosequencing

The group-specific primers were used to amplify earthworm DNA from 46 faecal samples produced by 46 different *P. augusta* individuals captured in the field prior to mining. The PCR protocol was the same as in Boyer *et al.* [25]. PCR products amplified from snail faeces were processed by electrophoresis (1.5% agarose gel) followed by a gel extraction and DNA purification (Qiagen Qiaquick© PCR gel extraction kit). The PCR products from all 46 samples were diluted to 0.5 ng/µl and pooled following the manufacturer's recommendation for amplicon sequencing with the *Roche Genome Sequencer FLX System*. One sixteenth of the full pyrosequencing plate was used. DNA reads obtained from 454-pyrosequencing were filtered to exclude amplicons with unexpected lengths (<120 bp or >160 bp) and amplicons lacking a complete primer. Unique amplicons were also discarded and only those that were detected at least 5 times were taken into account in the analysis, with the aim of filtering out chimeric sequences and PCR artefacts.

DNA reads were then compared to the earthworm DNA library (containing 15 species) using the BLAST program [30]. Reads that did not correspond to any species from the library were compared to the Genbank database using the BLASTn algorithm to confirm that they corresponded to earthworm DNA. If so, they were considered additional species.

## Results

### Bioinformatics

**Figure 1:**
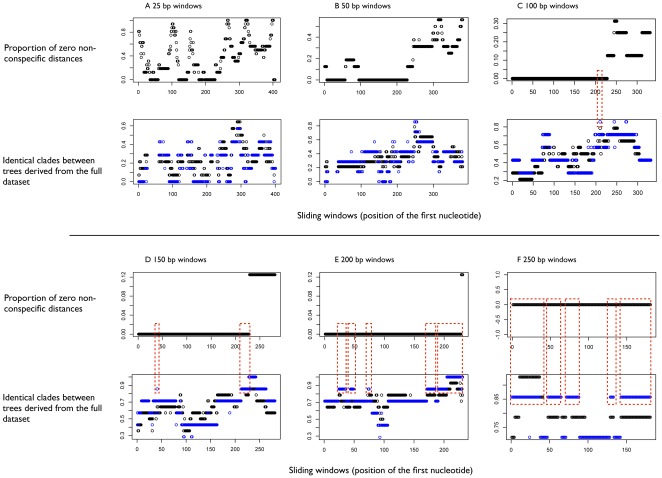
Sliding window analysis. The windows are 25, 50, 100, 150, 200 and 250 bp (from **A** to **F**). X axes: starting base of the window. Y axes on top graphs: proportion of zero non-conspecific distances. Y axes on bottom graphs: proportion of identical clades between trees derived from each window and trees derived from the full dataset. Black circles are based on all nodes and blue circles on shallow nodes only, i.e. nodes tipwards of the median node depth. The red boxes indicate the positions of highly informative windows.

The length of the selected window had a large impact on the identification success rate. With shorter windows, specimen identification was often not achievable because non-conspecific distances could be zero (Fig. 1). When longer windows were considered, more accuracy was observed, with many of the windows displaying no zero non-conspecific distances (i.e. 100% accuracy). Using longer windows also resulted in a better representation of the tree topology with higher proportions of clades identical to those obtained with the full 430 bp dataset.

According to the sliding window analysis, the shortest highly informative window was a 100 bp fragment starting at base 210 of the alignment (Fig. 1), which corresponds to position 11934 of the published *L. terrestris* mitochondrial genome sequence. This window provides accurate specimen identification (no zero non-conspecific distances) and a tree topology similar to that obtained from the full dataset (85.7% of the shallow clades are the same) (Fig. 1C).

### Pyrosequencing

**Figure 2:**
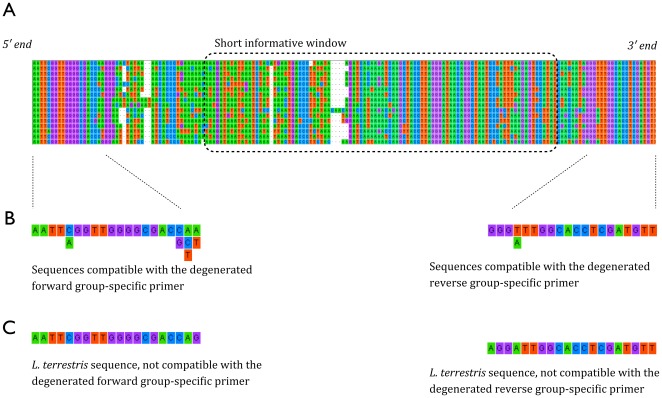
Position of the selected short informative window and the group-specific primers. For all sequences, the 5′ end is on the left and the 3′ end is on the right. Green=Adenine (A), orange=Thymine (T), blue=Cytosine (C), Purple=Guanine (G), White=gap. (**A**) Portion of the mitochondrial 16 S sequence from the 15 New Zealand endemic species comprising the DNA library (lines 1–15) and one *L. terrestris* (line 16). The represented portion covers positions 11890 to 12055 of the *L. terrestris* mitochondrial genome sequence [26]. (**B**) List of sequences compatible with the degenerate group-specific primers. When several nucleotides are compatible for a given position, they are written vertically, e.g. the first nucleotide of the Forward sequence must be A, the fifth nucleotide must be C or A. (**C**) Example of sequences not compatible with the degenerated group-specific primers (*L. terrestris*).

**Figure 3:**
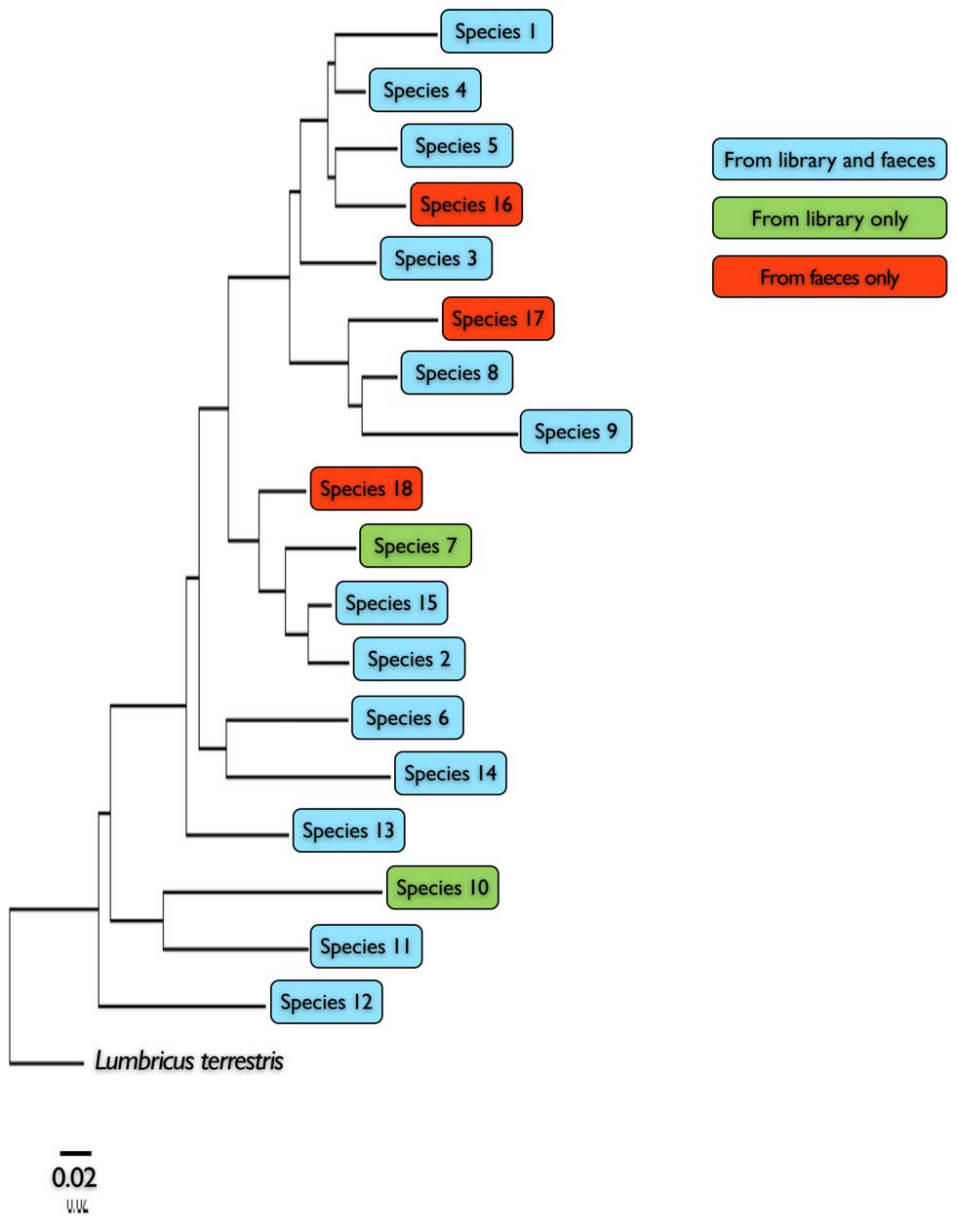
Neighbour-joining tree based on the 134 bp fragment of 16 S mitochondrial rDNA for 18 putative New Zealand endemic earthworms and one outgroup sequence (*L. terrestris*). Labels correspond to species in the DNA library that were found in snail faeces (blue), species in the DNA library that were not found in the snail faeces (green), and species that were not present in the DNA library but were found in the snail faeces (red). The tree is drawn to scale, with horizontal branch lengths corresponding to percentage differences (see scale for 2% difference).

Conserved regions were used to design group-specific primers that amplify a 134 bp region encompassing this 100 bp window (Fig. 2). The use of these group-specific primers, coupled with 454-pyrosequencing, led to the successful amplification and retrieval of 8,742 DNA sequences of which 8,712 (99.7%) were of good quality (i.e. of expected length and containing at least one primer). The number of sequences corresponding to the earthworm DNA library was 7,210 (83%). Earthworm DNA retrieved from snail faeces contained 13 species from the library. Only species 7 and 10 were not detected in the faecal samples (Fig. 3). The remaining sequences (17%), which had no correspondence in the DNA library, formed three distinct clades, which may correspond to three additional species (Fig. 3).

## Discussion

### The diet of *P. augusta*

Earthworm DNA that had been degraded through digestion by the carnivorous landsnail *P. augusta* was successfully amplified using group-specific primers designed to amplify the shortest (100 bp) possible informative window. Using a short section of a single gene to identify predated earthworm species was particularly effective because (1) likely prey species were restricted to a narrow taxonomic range and (2) data for most of the potential prey species had been generated and were available as a DNA reference library. Pyrosequencing of prey DNA obtained from faecal samples led to the detection of 18 earthworm species comprising the diet of *P. augusta*. Most of these species are likely to be new to science as the New Zealand earthworm fauna is still largely unknown [31], [32]. Sequences that did not match any references in the earthworm library (17%) formed three distinct clades. The corresponding amplicons had a minimum of 7.5% divergence from their closest sequence in the DNA library, which corresponds to 10 nucleotide differences and strongly suggests that they are three additional species. A BLAST analysis of these sequences showed that they were closely related to those of other New Zealand earthworms. These additional species are likely to be endemic species that were overlooked during the earthworm inventory [29], and probably correspond to ‘anecic’ earthworms: deep burrowing species that only come to the surface at night to feed on dead leaves and plant debris [33]. Such behaviour would make them accessible to the nocturnal snails but unlikely to be found in 20 cm deep soil samples collected during the day. Alternative explanations for these three amplicons include sequencing errors and the possible amplification of nuclear mitochondrial pseudogenes (NUMTs), which can be co-amplified with mitochondrial DNA paralogs [34] and can lead to false interpretations in molecular diet analyses [35], [36], particularly when short fragments of DNA are targeted [37]. However, NUMTs are probably less likely to be amplified than cytoplasmic mtDNA due to lower copy number, especially in degraded DNA samples and to our knowledge, NUMTs have not been reported for Annelida or Mollusca [38], [39]. Two of the amplicons regarded as potential new species (species 17 and 18) were detected 333 times across two samples and 505 times across two samples, respectively. These are therefore less likely to be the product of NUMTs or sequencing errors. However, the amplicon corresponding to species 16 was detected only seven times, and from only one faecal sample, which suggests either a rarely predated earthworm species or a possible sequencing error. These hypotheses could be tested with further data, especially in regard to locating the previously unobserved earthworms by sampling from deeper soil layers.

### Sequencing degraded DNA

When amplification of degraded DNA with conventional markers fails because DNA is broken down into short fragments, the alternative is to design new primers specifically to amplify a shorter region [40]. In this case, the short region must contain enough inter-specific variability to ensure accurate specimen identification. Short DNA regions, or mini-barcodes are becoming increasingly popular for the analysis of environmental DNA samples [10], [11], [15], [41]. Although a few studies have selected mini-barcodes based on objective criteria [42], [43], they focused on a limited number of candidates. The work described here appears to be the first to propose a method for comparing all possible mini-barcodes for a given molecular marker. We measured the performance of 1745 potential mini-barcodes compared to eight [42], 10 [43], 13 [44] and 26 [45], in other studies.

The sliding window analysis in the R package SPIDER [17] provides molecular ecologists with a way to objectively select the most informative region of the gene they aim to amplify. This method is applicable to any taxon or gene region and is capable of analysing reasonably sized alignments in a matter of seconds to a few minutes [17]. By using published sequences from online databases (e.g. BOLD or GenBank), the sliding window analysis can be applied at virtually no cost to determine which molecular marker will contain the smallest and most informative window appropriate for the group under study. This method also allows the users to set their own objectives in terms of accuracy of specimen identification and/or conformity to a given tree topology. Because traditional cut-off thresholds for full-length sequences are not directly applicable to shorter sequences [45], we considered only zero non-conspecific distances to be below the cut-off for species differentiation (i.e. a single nucleotide difference within the mini-barcode was considered sufficient for species diagnosis). Although such a liberal criterion could lead to false positives (i.e. individuals from the same species being categorised as different species), when using the selected 134 bp mini-barcode, differences between species were always >4% (Fig. 2), corresponding to at least five nucleotide differences making false positives less likely.

In addition to the sliding window criteria presented here (distance to non-conspecifics and topological similarity), there are other useful metrics for the determination of the best window. Such criteria include species monophyly, summed genetic distance, average GC content, and species-diagnostic nucleotides, all of which are appropriate methods for other applications and are implemented in the package SPIDER [17].

### Further applications

Identification of specimens from environmental samples or for diet analyses (from gut contents or faeces) requires that all the potentially present species be represented in a reference sequence library [11]. However, unknown species may also display variability within the same highly informative window as known species, in which case detection of non-inventoried species is possible. Primers should be restricted to a narrow taxonomic group, as environmental samples typically contain non-target DNA. Therefore, efficient group-specific primers are required, which is often not compatible with large-scale environmental barcoding data, from which diverse DNA assemblages are expected [46]. In such cases, a combination of several primer pairs may be necessary. Despite providing information on the best possible mini-barcode regions, the current version of SPIDER does not provide functions for designing of the actual primers; many other software packages are specifically designed for this purpose, e.g. *Qprimer* [47], *Green SCPrimer* [48], *Uniprime* [49], *ecoPrimers* [50], etc. However, the nucleotide diagnostics functions of SPIDER can be used to identify sites useful for group specific primer design, and can then be used together with other programs to fit primers around the best possible mini-barcode(s). Some of these programs also assess the suitability of mini-barcodes for specimen identification [43], [50] by selecting primer combinations, matching the amplicons in pairs and comparing the barcode resolution capacity of each pair to decide which is best for identification purposes [43]. Our approach is different in that it focuses on identification success and information content over primer design by comparing all possible mini-barcodes for a given molecular marker and set of taxa.

Pyrosequencing is ideal for the analysis of mixed DNA samples. It represents a significant improvement in molecular diet analyses, because (1) it supplants non-sequencing methods, such as denaturing gradient gel electrophoresis, which requires additional gel extraction and sequencing for identification of prey species [51], (2) it does not require the design of a different pair of primers for each potential prey species, or the use of multiplex PCR reactions [52], and (3) all prey species are detected and sequenced simultaneously without the need for cloning [36] so that increasing the number of samples or the number of potential prey species has little impact on the cost of the analysis. However, because of the technical limitations, pyrosequencing is currently limited to short DNA fragments (<200 bp for the Roche Genome sequencer FLX System used here). As a result, pyrosequencing analyses have mostly been used along with powerful bioinformatic tools that detect overlapping regions and splice together small fragments of the genome [53]. When using a sliding window analysis *a priori*, the information content of short length sequences can be assessed, with the aim of negating the requirement for additional bioinformatic sequence consolidation of several longer, less informative regions.

Our method is also relevant for the molecular analysis of historical or ancient DNA (e.g. old museum specimens, palaeo-samples) for which DNA amplification and sequencing often fails with conventional primers [6]. Previous research has highlighted the compatibility of the pyrosequencing approach with barcoding of historical specimens [54]. When combined with techniques such as whole-genome amplification (e.g. Genomiphi®), this approach has the potential to constitute an improved option for sequencing highly degraded DNA.

Another important application lies in the search for alternatives to the barcoding region. Although COI is widely used for specimen identification in many animals, it appears to be unsuitable for certain groups, such as Anthozoa [55], [56], plants [57], fungi [58], [59] and protists [60]. Sliding window analysis offers an objective method for comparing molecular markers proposed by different authors in these difficult situations. For taxa where no obvious barcode has been proposed, sliding window analysis can greatly help in evaluating the best candidates.
